# The transcriptional landscape of dorsal root ganglia after sciatic nerve transection

**DOI:** 10.1038/srep16888

**Published:** 2015-11-18

**Authors:** Shiying Li, Chengbin Xue, Ying Yuan, Ruirui Zhang, Yaxian Wang, Yongjun Wang, Bin Yu, Jie Liu, Fei Ding, Yuming Yang, Xiaosong Gu

**Affiliations:** 1 Jiangsu Key Laboratory of Neuroregeneration, Co-innovation Center of Neuroregeneration, Nantong University, Nantong, JS 226001, China

## Abstract

Following peripheral nerve injury, transcriptional responses are orchestrated to regulate the expression of numerous genes in the lesioned nerve, thus activating the intrinsic regeneration program. To better understand the molecular regulation of peripheral nerve regeneration, we aimed at investigating the transcriptional landscape of dorsal root ganglia (DRGs) after sciatic nerve transection in rats. The cDNA microarray analysis was used to identify thousands of genes that were differentially expressed at different time points post nerve injury (PNI). The results from Euclidean distance matrix, principal component analysis, and hierarchical clustering indicated that 2 nodal transitions in temporal gene expressions could segregate 3 distinct transcriptional phases within the period of 14 d PNI. The 3 phases were designated as “a stress response phase”, “a pre-regeneration phase”, and “a regeneration phase”, respectively, by referring to morphological observation of post-nerve-injury changes. The gene ontology (GO) analysis revealed the distinct features of biological process, cellular component, and molecular function at each transcriptional phase. Moreover, Ingenuity Pathway Analysis suggested that differentially expressed genes, mainly transcription factors and genes associated with neurite/axon growth, might be integrated into regulatory networks to mediate the regulation of peripheral nerve regeneration in a highly cooperative manner.

Although adult mammalian peripheral nerves have an intrinsic ability to spontaneously regenerate after transection, the functional outcomes of peripheral nerve repair are often unsatisfactory even with the help of currently available therapies^1^,^2^,^3^. Neuroscientists and clinicians have been striving to gain more detailed insights into molecular mechanisms underlying peripheral nerve regeneration and to develop more effective therapeutic approaches to peripheral nerve injury. Following axonal transection, a series of pathophysiological events occurs in the lesioned tissues, and highly orchestrated gene expression programs are activated, accompanied by phenotypic and functional changes of neural and non-neural cells^4^. Previous studies have examined time-dependent expression changes of many genes in proximal and distal peripheral nerves after nerve injury by using cDNA array-based expression analysis that allows the identification of a diverse spectrum of genes that are differentially expressed between normal and disease conditions^4^,^5^,^6^,^7^,^8^,^9^. Despite high-throughput gene data obtained in these studies, more comprehensive analysis remains to be done by the aid of newly-developed statistical and bioinformatic tools for acquiring valuable information about molecular regulation of transcriptional responses of the peripheral nervous system to traumatic injury.

Sciatic nerve injury is a commonly used model for peripheral nerve regeneration studies, and sensory neurons extending into the sciatic nerve are located in the L4–L6 dorsal root ganglia (DRGs). Once primary sensory neurons are primed by peripheral axonal injury, they are likely to grow more rapidly in response to a subsequent lesion, which has been known as the conditioning effect^10^,^11^. Peripheral axonal injury triggers axonal growth from DRG neurons, conditions neurons to grow extensively, and facilitates peripheral nerve regeneration^12^. Several molecular mediators in DRGs have been identified to play regulatory roles in peripheral nerve regeneration^13^,^14^,^15^,^16^,^17^,^18^,^19^,^20^, but more comprehensive studies are still required to obtain a global perspective of how these molecular mediators, coupled with the related bioprocesses and signaling pathways, are deliberately orchestrated to activate the intrinsic regenerative programs of peripheral nerves. The aim of this study was to investigate the transcriptional landscape of rat DRGs in response to sciatic nerve transection. The cDNA microarray analysis showed that thousands of genes were differentially expressed at different time points following sciatic nerve transection. Various bioinformatic tools were subsequently used to analyze the microarray-derived data sets, revealing some interesting aspects about gene regulation of peripheral nerve regeneration.

## Results

### Global overview of differentially expressed genes in rat DRGs following sciatic nerve transection

**Figure 1:**
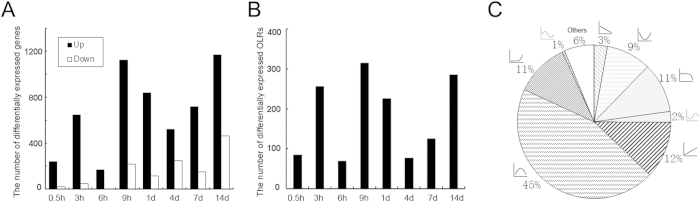
Differentially expressed genes in DRGs of rats after sciatic nerve transection. Histogram (**A**) showing the number of up-regulated genes or down-regulated genes at different time points post nerve injury (PNI) as compared to control, respectively. Here the control refers to animals that were killed by cervical dislocation immediately after they received sham surgery (at 0 h PNI). Histogram (**B**) showing the number of differentially expressed olfactory receptors at different time points post nerve injury (PNI) as compared to control. (**C**) Pie chart showing the number ratio (in percent) of differentially expressed genes involved in each of 9 expression profiles to the total differentially expressed genes.

The cDNA microarray analysis was performed on the RNA sample extracted from L4-L6 DRGs of rats at different time points following sciatic nerve transection. All data sets were obtained from 3 independent experiments to ensure reproducibility. A huge number of genes showed the differential expression in DRGs at different time points post nerve injury (PNI), and the number of up-regulated genes seemed to be significantly larger than that of down-regulated genes, suggesting the activation of most genes in response to peripheral nerve injury. The number of up-regulated and down-regulated genes changed with time during the period of 0.5 h to 14 d PNI, and these changes are illustrated in Fig. 1A. Meanwhile, all differentially expressed genes at indicated time points are listed in Table S1.

Surprisingly and interestingly, a large number (1111) olfactory receptors were present in rat DRGs. Out of them, 510 were differentially expressed following sciatic nerve transection (Table S2), and 505 olfactory receptors were up-regulated (Fig. 1B, Table S2).

All the differentially expressed genes showed 9 distinct expression profiles over the 14 d period PNI (Table S3). Out of the total number of differentially expressed genes, 45% (the majority) were first up-regulated to reach a peak and then recovered in a manner resembling an inverted U-shape; 9% were first down-regulated to reach a peak and then recovered in a manner resembling a U-shape; 12% were linearly up-regulated; 3% were linearly down-regulated; 11% were kept unchanged at 0.5 h to 4 d PNI and then down-regulated until 14 d PNI, and another 11% were kept unchanged at 0.5 h to 4 d PNI and then up-regulated until 14 d PNI (Fig. 1C, Table S3).

### Three transcriptional phases of peripheral nerve regeneration

**Figure 2:**
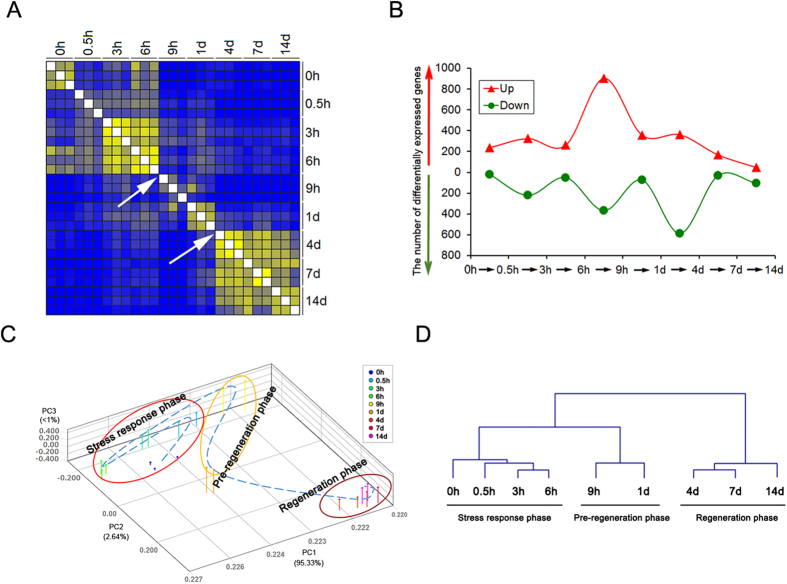
Three distinct transcriptional phases after peripheral nerve injury. (**A**) Euclidean distance heatmap between different time points post nerve injury (PNI), as labeled on top and right margins. Arrows indicate 2 nodal transitions. (**B**) The number of up-regulated (▲) or down-regulated (●) genes at each time point PNI as compared to that at previous time point PNI. (**C**) Principal component analysis of differentially expressed genes at different time points PNI. (**D**) Dendrogram of gene expression profiles, as created by hierarchical clustering analysis. The 2 nodal transitions in temporal gene expressions could segregate 3 distinct transcriptional phases within the period of 14 d PNI. For the sake of convenient description, these 3 phases were arbitrarily designated as “a stress response phase”, “a pre-regeneration phase”, and “a regeneration phase”, respectively.

We used a Euclidean distance matrix to visualize the magnitude of transcriptional changes in DRGs after sciatic nerve transection. The population of differentially expressed genes exhibited 2 nodal transitions between 6 and 9 h PNI and between 1 and 4 d PNI, respectively (Fig. 2A). The number of up-regulated or down-regulated genes reached the highest value during the period of 6 to 9 h PNI or 1 to 4 d PNI, respectively (Fig. 2B, Table S4). Principal component analysis (PCA) also allowed us to segregate 3 distinct phases within the period of 14 d PNI just using the 2 transitions as boundaries between phases (Fig. 2C). For the sake of convenient description, these 3 phases were arbitrarily designated as “a stress response phase”, “a pre-regeneration phase”, and “a regeneration phase”, respectively, by referring to morphological observation of post-nerve-injury changes^21^,^22^. A dendrogram created by hierarchical clustering (Fig. 2D) provided further evidence for the existence of 3 phases. Moreover, the 3 transcriptional phases were sequentially linked together.

### Biological process analysis

**Figure 3:**
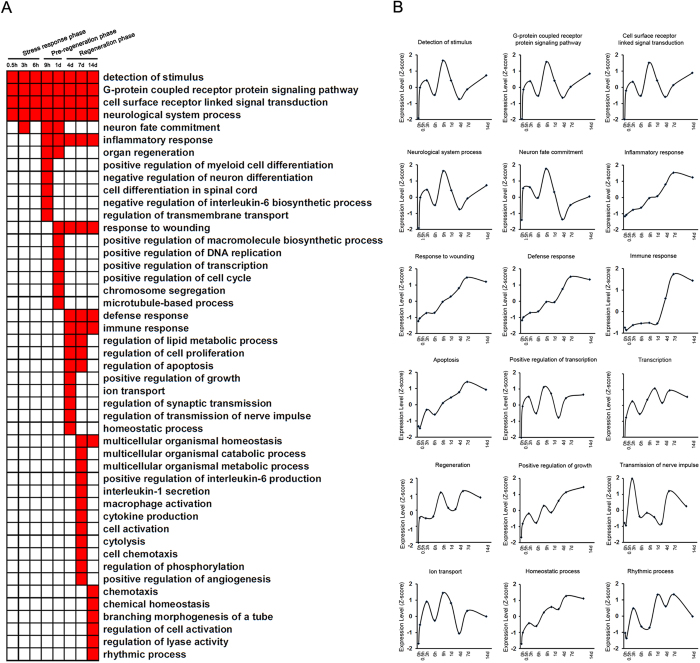
Biological process analysis of differentially expressed genes. (**A**) The enriched categories (red color) of biological processes at different time points post nerve injury (PNI). (**B**) The average expression profiles of differentially expressed genes, which were involved in major biological processes, in DRGs at different time points PNI.

Biological processes related to differentially expressed genes were identified by gene ontology (GO) analysis and listed in Table S3. The enriched categories of biological processes at different time points PNI were labeled in Figure 3A. During the stress response phase, such categories as detection of stimulus, signaling transduction (G-protein coupled receptor protein signaling pathway and cell surface receptor linked signal transduction), and neurological system process were enriched. During the pre-regeneration phase, such biological process categories as DNA replication and transcription (positive regulation of macromolecule biosynthetic process, positive regulation of DNA replication, and positive regulation of transcription) were enriched, and other biological process categories, such as inflammatory response, negative regulation of interleukin-6 biosynthesis, etc. were also enriched. During the regeneration phase, such biological process categories as cell proliferation, growth, apoptosis, and activation (regulation of cell proliferation, positive regulation of growth, regulation of apoptosis, cell activation, and regulation of cell activation) were enriched, and other biological process categories, such as immune response, positive regulation of interleukin-6 production, macrophage activation, cytokine production, ion transport, homeostatic process, and rhythmic process, etc. were also enriched.

The 12 biological processes became the focus of our attention, which included detection of stimulus, G-protein coupled receptor protein signaling pathway, cell surface receptor linked signal transduction, neurological system process, neuron fate commitment, inflammatory response, response to wounding, defense response, immune response, ion transport, regulation of apoptosis, and rhythmic processe. The genes were grouped according to their involvement to these 12 biological processes (Table S6). In order to analyze time-dependent changes of biological processes, the average expression profiles of differentially expressed genes involved in each biological process were examined (Fig. 3B). Several major biological processes are briefly described below.

1. Detection of stimulus. Detection of stimulus is a biological process, in which injury stimulus is detected and converted into a molecular signal. The average expression profiles of differentially expressed genes involved in this process showed a curved change with 2 peaks and 2 troughs alternately appearing at 3, 6, 9 h and 4 d PNI, respectively (Fig. 3B). The differentially expressed genes involved in detection of stimulus included many olfactory receptors as well as AIPL1, CHRNA10, GJA10, GNAT1, PDE6B, TAS2R16, etc (Table S6.1).
2. Signaling transduction. Signaling pathways were activated following detection of stimulus, and two major signaling pathways were G-protein coupled receptor protein signaling pathway and cell surface receptor linked signal transduction. The average expression profiles of differentially expressed genes involved in these two processes, just like those involved in detection of stimulus, exhibited a curved change with alternate peaks and troughs (Fig. 3B). The genes involved in these two biological processes included many olfactory receptors as well as TAS2Rs, GPRs, GABAs, CCRs, and TAARs, etc. (Table S6.2 and S6.3).
3. Response to stimulus. Response to wounding, defense response, inflammatory response, immune response, and apoptosis can be categorized into a big class called “response to stimulus”. The average expression profiles of differentially expressed genes involved in all these processes showed a sustained increase with a common peak at 7 d PNI (Fig. 3B). The differential expressed genes involved in these processes were listed in Table S6.4–S6.8.
4. Transcription. The average expression profiles of differentially expressed genes involved in two processes of positive regulation of transcription and transcription showed a curved change with a maximum appearing at 9 h PNI or 1 d PNI for these two processes respectively (Fig. 3B), The differential expressed genes involved in two processes were listed in Table S6.9–S6.10.
5. Regeneration and growth. The average expression profiles of differentially expressed genes involved in two processes of regeneration and positive regulation of growth showed a total increase with slight up and down changes (Fig. 3B). The differential expressed genes involved in two processes were listed in Table S6.11–S6.12.
6. Other biological processes. The average expression profiles of differentially expressed genes involved in other biological processes (neurological system process, neuron fate commitment, ion transport, rhythmic process) exhibited a curved change (Fig. 3B), and the corresponding genes were listed in Table S6.13–S6.18.

### Cellular component analysis

**Figure 4:**
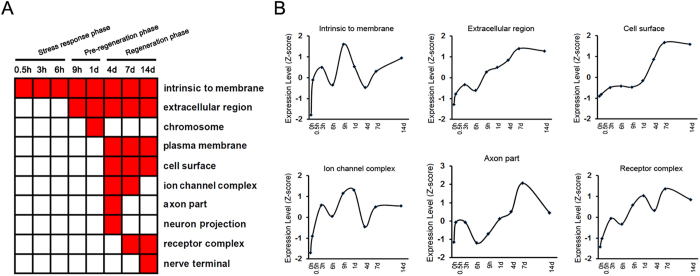
Cellular component analysis of differentially expressed genes. (**A**) The enriched categories (red color) of cellular components at different time points post nerve injury (PNI). (**B**) The average expression profiles of differentially expressed genes, which were involved in major cellular components, in DRGs at different time points PNI.

Cellular component annotations of differentially expressed genes were also accomplished by GO analysis (Table S7). The categories of cell components were enriched at different time points PNI (Fig. 4A). Interestingly, cellular component categories, such as intrinsic to membrane, chromosome, and axon part were just enriched during the stress response phase, “a pre-regeneration phase”, and “a regeneration phase”, respectively. The average expression profiles of differentially expressed genes involved in different cellular components also displayed time-dependent dynamic changes, each exhibiting their own distinct features (Fig. 4B, Table S8).

### Molecular function analysis

**Figure 5:**
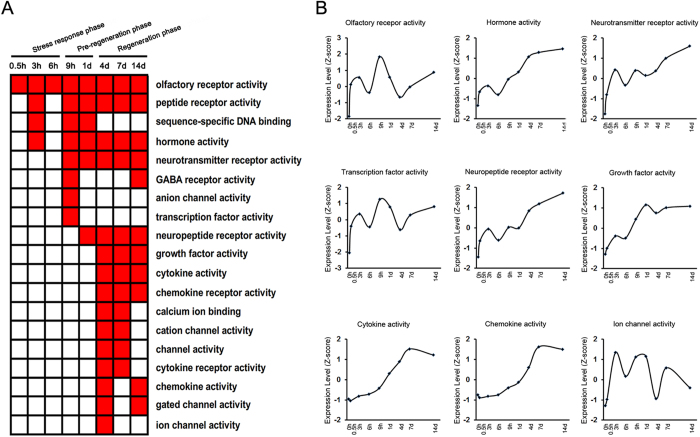
Molecular function analysis of differentially expressed genes. (**A**) The enriched categories (red color) of molecular functions at different time points post nerve injury (PNI). (**B**) The average expression profiles of differentially expressed genes, which were involved in major molecular functions, in DRGs at different time points PNI.

Molecular functions related to differentially expressed genes were also examined by GO analysis (Table S9). The categories of molecular functions were enriched at different time points PNI (Fig. 5A). During the stress response phase, such molecular function categories as olfactory receptor activity, peptide receptor activity, and hormone activity seemed to be enriched. During the pre-regeneration phase, transcription factor activity seemed to be enriched. During the regeneration phase, such molecular function categories as growth factor activity, cytokine activity, and ion channel activity, etc. seemed to be enriched. Certainly, further experimental evidence is needed for this analysis. The average expression profiles of differentially expressed genes involved in 9 molecular functions, including olfactory receptor activity, hormone activity, neurotransmitter receptor activity, transcription factor activity, neuropeptide receptor activity, growth factor activity, cytokine activity, chemokine activity, and ion channel activity, was investigated (Fig. 5B, Table S10). The average expression profiles of differentially expressed genes involved in olfactory receptor activity, transcription factor activity, and ion channel activity exhibited a curved change with the maximum appearing at 9, 9, and 3 h PNI, respectively. The profiles involved in other molecular functions showed a sustained increase during the 14 d period PNI.

### Cascade regulation of transcription factors

**Figure 6:**
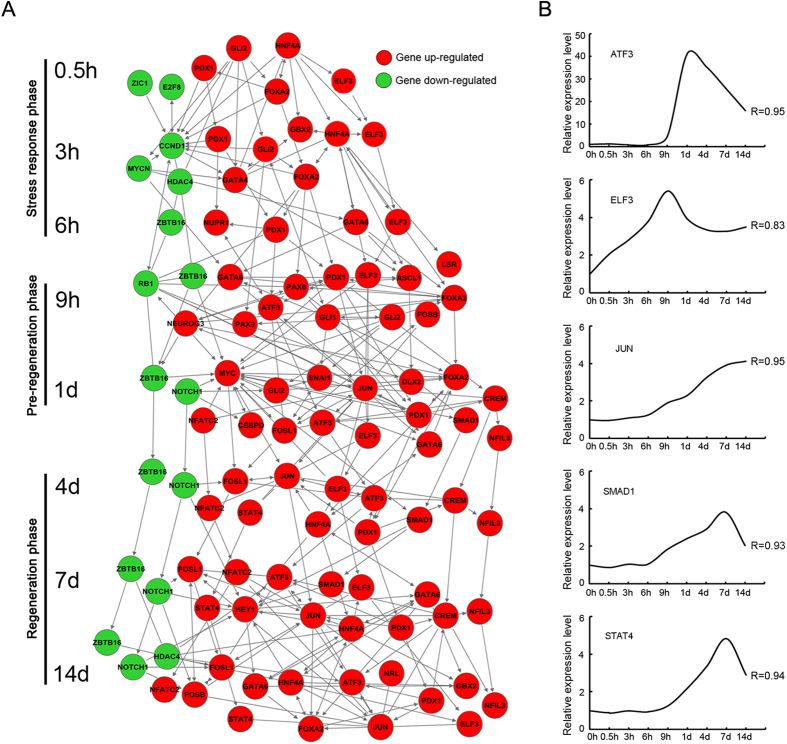
Cascade regulation of transcription factors. (**A**) Networks showing the connections and interactions between up-regulated (red) and down-regulated (green) transcription factors. (**B**) RT-qPCR validation of microarray analysis for the differential expression of several key transcription factors. R stands for the correlation coefficient between microarray and RT-qPCR data.

The transcription factors play an important role in the regulation of gene expression. In this study, the intersection of the rat transcription factors with the differentially expressed genes allowed us to identify the differentially expressed transcription factors. Then, the regulatory networks among the differentially expressed transcription factors were built by the aid of Ingenuity Pathway Analysis (IPA) (Fig. 6A). Figure 6A showed that the regulatory networks at 9 h and 1 d PNI (“a pre-regeneration phase”) seemed to be more complicated than those at other time points. And some genes, such as PDX1 GLI2, HNF4A, FOXA2, ELF3 opened up the cascade regulation from 0.5 h, and some genes, such as ATF3, JUN, SMAD1, ELF3, and STAT4, etc. played a hub role in the regulatory networks.

During the period of 0.5 h–14 d PNI, ATF3, JUN, SMAD1, ELF3, and STAT4 were considered as the key or hub genes in all transcription factors investigated here. Therefore, their differential expressions in DRGs after sciatic nerve transection were subjected to real-time quantitative reverse transcription (RT-qPCR) validation. RT-qPCR data were highly correlated to those from microarray analysis (Fig. 6B).

### Dynamic changes of differentially expressed genes associated with neurite/axon growth

The promotion or inhibition of neurite/axon growth determines the outcomes of peripheral nerve regeneration. Therefore we paid special attention to the differential expression of the genes that were closely associated with neurite/axon growth, and we were also concerned with the regulatory networks linking these genes.

**Figure 7:**
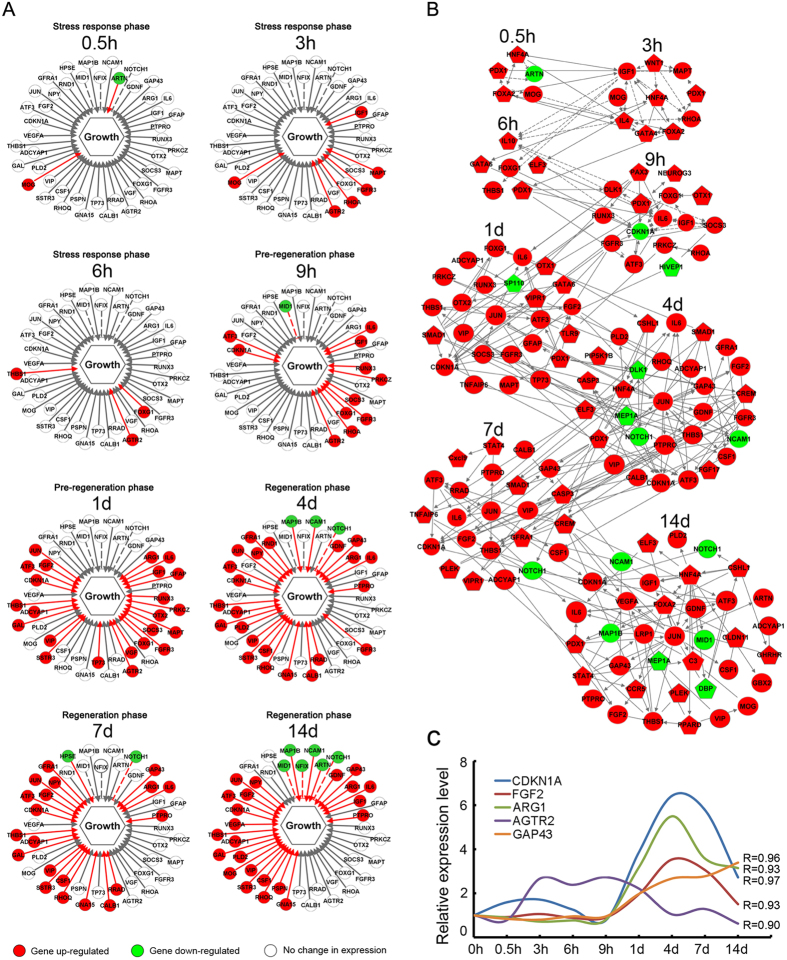
Dynamic changes of differentially expressed genes associated with neurite/axon growth. (**A**) Up-regulation (red), down-regulation (green) and unchange (no color) of genes associated with neurite/axon growth at different time points post nerve injury (PNI), where solid lines and dashed lines represent the activating and inhibitory roles of the corresponding genes on neurite/axon growth respectively. (**B**) The networks showing the connections and interactions among differential expressions (up-regulation labeled by red and down-regulation labeled by green) of genes associated with neurite/axon growth (circle) and their upstream regulatory genes (pentagon). (C) RT-qPCR validation of microarray analysis for the differential expression of several genes associated with neurite/axon growth. R stands for the correlation coefficient between microarray and RT-qPCR data.

By using IPA, the differentially expressed genes associated with neurite/axon growth were identified. Then, the issue of whether these genes promoted or inhibited neurite/axon growth was further examined, and the differential expression of these genes at different time points PNI was highlighted to determine how they were correlated to 3 transcriptional phases (Fig. 7A). During the stress response phase, a relatively small number of genes associated with neurite/axon growth was up-regulated. During the pre-regeneration phase”, the up-regulated genes associated with neurite/axon growth increased in number. They were ATF3, AGTR2, ARG1, CDKN1A, FGF2, IL6, IGF1, FGFR3, RUNX3, JUN, and SOCS3, etc., and their up-regulation might account for the triggering of “a pre-regeneration phase” and transmission to “a regeneration phase”. During the regeneration phase, the number of the up-regulated genes associated with neurite/axon growth further increased. Besides those genes occurring in “a pre-regeneration phase”, new ones were identified, which included GAP43, NPY, PLD2, GNA15, CSF1, RHOQ and PRAD, etc.

Cascade regulation of differentially expressed genes associated with neurite/axon growth was further analyzed. Most of them were up-regulated at different time points PNI, and their interconnections and interactions across time points constituted the complicated networks (Fig. 7B). Of note, inflammatory factors, such as IL4, IL10 and IL6, and several key transcription factors, such as JUN, ATF3, PDX1, ELF3, and HNF4A, play critical activating roles in the cascade amplification processes.

Based on their leading role in the promotion of peripheral nerve regeneration, the genes of AGTR2, ARG1, CDKN1A, FGF2, and GAP43 were subjected to RT-qPCR validation for their differential expressions at the PNI period, and RT-qPCR and microarray data were highly correlated to each other (Fig. 7C).

## Discussion

Peripheral nerve regeneration consists of a series of complicated cellular events and molecular pathways, driven by differential expression of a tremendous number of genes after peripheral nerve injury, and so the transcriptional landscape of dorsal root ganglia after sciatic nerve transection will be used to help understand the molecular regulation patterns of peripheral nerve regeneration.

As previously reported, the number of differentially expressed (mainly up-regulated) genes in proximal nerves continuously increased with the PNI time^7^. In contrast, the number of differentially expressed (mainly up-regulated) genes in DRGs exhibited a curved change with the PNI time, alternately increasing and decreasing over the period. The different time-dependent changes in the number of up-regulated expressed genes between in proximal nerves and in DRGs might be ascribed to their different neurohistological structures, suggesting the possible existence of relaxation and feedback processes in the response of DRGs to nerve injury. Similarly, a large number of olfactory receptors were found to be present in DRGs, just like in proximal nerves. Activated olfactory receptors play an initial role in a signal transduction cascade, ultimately producing a nerve impulse to be transmitted to the brain^23^,^24^,^25^. The differential expression of olfactory receptors in DRGs after sciatic nerve transection sparked our speculation about the possible regulatory role of olfactory receptors in peripheral nerve regeneration.

Euclidean distance calculation, principal component analysis (PCA), and hierarchical clustering, helped us to divide the period of 14 d PNI into 3 transcriptionally distinct phases. Previous studies^22^ have proposed that there exist different stages after peripheral nerve injury, for example, roughly two stages (early and later stages separated by 3 d PNI). This point of view is derived mainly from a morphological perspective, while our finding about 3 transcriptional phases represents an opinion based on molecular-level understanding of injury and regeneration in DRGs after peripheral nerve injury. The linkage and transition at two nodes between 3 transcriptional phases might provide useful information about how to improve peripheral nerve regeneration by identifying regulator genes at different stages and modulating gene accessibility to the necessary transcription machinery.

We used GO analysis to organize all differentially expressed genes in DRGs after sciatic nerve transection into hierarchical categories according to biological processes, cellular components, and molecular functions. We were interested in which categories of biological processes were enriched at different time points PNI, and these molecular-level investigations would at least make clear the understanding of behavior modulations of DRGs after sciatic nerve transection on the basis of morphological observation. The average expression profiles of differentially expressed genes in the biological processes of detection of stimulus or signaling transduction displayed a curved change rather than a continuous increase over the 14 d period. In contrast, the gene expression profile in processes of response to stimulus (including inflammatory response, response to wounding, defense response, immune response, and apoptosis) exhibited a continuous increase with a maximum at 7 d PNI.

Peripheral nerve injury may cause inflammation in DRGs through a complex series of molecular and cellular mechanisms^26^, and we found that GO biological process category of inflammatory response was significantly enriched from 9 h until 14 d PNI. Inflammation usually has pleiotropic effects on nerve regeneration^27^. On one hand, excessive inflammation produces detrimental effects on nerve regeneration^27^,^28^, which might account for the enrichment of negative regulation of IL6 biosynthetic process at 9 h PNI. On the other hand, inflammation can play a positive role in nerve regeneration^26^,^29^,^30^,^31^, which might be responsible for the enrichment of positive regulation of IL6 production at 7 d PNI (“a regeneration phase”). Moreover, diverse immune responses, including IL-1 secretion, macrophage activation, and cytokine production, were also enriched during a regeneration phase. All these results were in agreement with previous findings regarding the critical regulation of peripheral nerve regeneration by neuroimmune pathways^30^,^32^,^33^,^34^.

GO categories of cellular component and molecular function were further ascribed to differentially expressed genes, providing some interesting information for transcriptional changes after peripheral nerve injury. The information was, to a large degree, consistent with that derived from GO biological process annotations. Starting from 0.5 h PNI (“a stress response phase”), the enrichment of differentially expressed genes in cellular component and molecular function GO categories was in “intrinsic to membrane” and “olfactory receptor activity”, respectively. These categories were well matched to biological process categories enriched also from 0.5 h PNI, such as “detection of stimulus” and “signaling transduction”. The biological process categories of regulation of DNA replication or transcription were enriched during “a pre-regeneration phase”, and meanwhile the enriched cellular component and molecular function GO terms were “chromesome” and “transcription factor activity”, respectively. During “a regeneration phase”, the enriched biological process categories (e.g. regulation of cell proliferation, growth, apoptosis) were linked to the enriched cellular component categories (e.g. axon part) and molecular function categories (e.g. growth factor activity) terms.

A host of transcription factors have been implicated in DRGs responses to peripheral nerve injury, and they are integrated by intracellular signaling networks^35^,^36^. In this study, we examined cascade regulation of transcription factors. These differential expressions of transcription factors were interconnected and cascade-transmitted to form complex networks and play regulatory roles within 3 distinct phases after peripheral nerve injury. ATF3, JUN, SMAD1, and STAT4 were up-regulated while NOTCH1 was down-regulated throughout the period from “a pre-regeneration” to “a regeneration phase”. Evidence shows that ATF3, JUN, SMAD1 and STAT4 are neuronal regeneration-enhancing factors^12^,^35^,^37^,^38^, but NOTCH1 neuronal regeneration-limiting ones^39^. Accordingly, the results of our study supported these previous findings.

The essence of peripheral nerve regeneration is the outgrowth of neurites/axons from lesioned neurons, and so we further investigated the regulation of genes associated with neurite/axon growth. During the stress response phase, the regrowth of neurites/axons remained to be induced which was indicated by the small number of genes associated with neurite/axon growth that showed increased expression levels. From “a pre-regeneration phase” to “a regeneration phase”, however, an increasing number of genes associated with neurite/axon growth were gradually up-regulated, which included ATF3, CDKN1A, IL6, IGF1, FGFR3, RUNX3, JUN, SOCS3 and AGTR2, NPY, PLD2, GNA15, GAP43, CSF1, RHOQ and PRAD, etc. Importantly, these genes have proven to be useful in promoting nerve regeneration.^12^,^37^,^40^,^41^,^42^,^43^,^44^,^45^,^46^.

**Figure 8:**
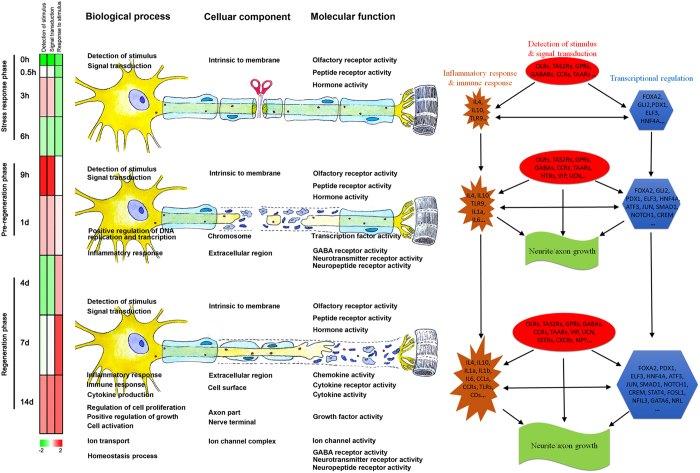
Schematic diagram illustrating the global view of transcriptional changes in DRGs after sciatic nerve transection. Bioinformative analysis showing the 3 distinct transcriptional phases within the time period of 0 h–14 d (left part); GO analysis displaying the category enrichments of some representative biological processes, cellular components, and molecular functions at each transcriptional phase, and also shown is a schematic illustration of morphological alterations occurring in the lesioned nerve after sciatic nerve transection (central part); IPA analysis revealing that the differentially expressed genes involved in detection of stimulus, signal transduction, inflammatory response, immune response, and transcriptional regulation could be linked to each other to mediate the regulation of neurite/axon growth in a highly cooperative manner (right part).

Finally, a schematic diagram is presented to illustrate all data and analyses in this study (Fig. 8). According to the results from Euclidean distance matrix, principal component analysis, and hierarchical clustering, the time period of 0 h–14 d PNI could be divided into 3 distinct transcriptional phases, and the ensuing GO analysis fully displayed the distinct features of biological process, cellular component, and molecular function at each transcriptional phase, verifying our artificial designation about 3 transcriptional phases. The average expression profiles of differentially expressed genes in the biological processes of detection of stimulus or signaling transduction displayed a curved change over the 14 d period; in contrast, the gene expression profile in processes of response to stimulus exhibited a continuous increase with a maximum at 7 d PNI, suggesting the possible existence of relaxation and feedback processes in the response of DRGs to nerve injury. IPA analysis revealed that the differentially expressed genes involved in detection of stimulus, signal transduction, inflammatory response, immune response, and transcriptional regulation could be linked to each other to mediate the regulation of neurite/axon growth in a highly cooperative manner. For example, two categories of detection of stimulus and signal transduction were enriched immediately following nerve injury, and then they were transmitted to two other categories of inflammatory response and immune response and also to another category of transcription, which 3 categories were reciprocally influenced and further transmitted to a category of neurite/axon growth through cascade amplification.

## Methods

### Animal surgery and sample collection

Adult male Sprague-Dawley (SD) rats (180–220 g) were obtained from the Experimental Animal Center of Nantong University). All experimental procedures involving animals were carried out according to the institutional animal care and use guidelines of Nantong University and approved ethically by the Administration Committee of Experimental Animals, Jiangsu Province, China. All animals underwent surgery for transection of the left sciatic nerve (creating a 10 mm long defect) according to well-established protocols^7^,^47^, and they were randomized into a control group and 9 groups according to different time points (0, 0.5, 3, 6, and 9 h, and 1, 4, 7, and 14 d) PNI. The animals receiving a sham surgery constituted the control group (0 h PNI). The L4-6 DRGs were harvested from animals in different groups after they were killed by cervical dislocation at the corresponding time points PNI.

### Microarray hybridization and analysis

Total RNA was extracted from DRG samples with Trizol kit (Life technologies, Carlsbad, CA) as per the manufacturer’s protocols. The quality of purified RNA was evaluated (RIN≥6) by a bioanalyzer 2100 (Agilent technologies, Santa Clara, CA). Qualified total RNA was amplified and labeled with a Low Input Quick Amp Labeling Kit (Agilent technologies). After further purification with RNeasy Mini kit (Qiagen, Valencia, CA), the labeled cDNA was quantified by determining the absorbance at 260 nm with a Nanodrop ND1000 spectrophotometer (NanoDrop Technologies, Wilmington, DE), and then hybridized to microarray slides under 60 °C for 17 h with a gene expression hybridization kit (Agilent Technologies). The slides were analyzed by a microarray scanner (Agilent Technologies), and Agilent feature extraction software was used to read out the microarray images. Each sample was tested in triplicates. All microarray experiments were conducted in National Engineering Center for Biochips at Shanghai, China.

Array normalization and error detection were performed with Silicon Genetics’ GeneSpring GX Version 10.0 (Agilent Technologies) via the enhanced Agilent feature extraction import preprocessor. All data was MIAME compliant, and the raw data were submitted to the MIAME compliant database (NCBI Accession number: Series GSE30165, GSE65053). The values of poor quality intensity and low dependability were removed according to a “filter on flags” feature, where standardized software algorithms determined which spots were “present”, “marginal”, or “absent”. Filters were set to retain only the present and marginal values. Microarray data were normalized with algorithms supplied with the feature extraction software, and independent sample t-tests were performed for comparisons between each group and control group unless otherwise stated (for example, in Fig. 2B). A p value of less than 0.05 and a mean expression change higher than 2 fold was considered statistically significant for further analysis.

### Euclidean distance calculation, PCA and hierarchical clustering

Euclidean distance calculation, PCA, and hierarchical clustering were performed on log2-transformed mean-centered datasets as described previously^48^,^49^. In brief, Euclidean distance heatmap was created with the HeatMapImage GenePattern module. PCA was performed with “Population PCA” tool (http://cbdm.hms.harvard.edu/LabMembersPges/SD.html). Hierarchical clustering was performed with the HierarchicalClustering module from GenePattern, utilizing Euclidean distance, and was visualized with the HierarchicalClusteringViewer GenePattern module.

### GO analysis

GO terms were used to describe 3 attributes of differentially expressed genes: the biological process, cellular component, and molecular function of these genes. Analysis of GO categories enrichment was performed with DAVID tools^50^,^51^, and then the average expression profiles were calculated as described previously^52^,^53^.

### Regulatory network analysis

Ingenuity Pathway Analysis (IPA) (http://ingenuity.com/index.html, Ingenuity Systems, Redwood City, CA) on-line software was used to intersect transcription factors or genes associated with neurite/axon growth with the differentially expressed genes, thus identifying the differentially expressed transcription genes or genes associated with neurite/axon growth^54^. And the interconnections and interactions among these genes were further examined, thus creating regulatory networks and modeling signaling pathways. A network is a graphical representation of the molecular relationships between molecules. Molecules are represented as nodes, and the biological relationship between two nodes is represented as an edge (line). All edges are supported by at least 1 reference from the literature, from textbook, or from canonical information stored in the Ingenuity® Knowledge Base. Human, mouse, and rat orthologs of a gene are stored as separate objects in the Ingenuity® Knowledge Base but are represented as a single node in the network. To create connections between molecules, the Build – Connect tool of IPA was used, with direct (solid line) or indirect (dot line) relationships shown.

### RT-qPCR

The same RNA samples as used in microarray analysis were subjected to RT-qPCR for determination of the mRNA expression of some genes, such as ATF3, JUN, SMAD1, ELF3, STAT4, CDKN1A, FGF2, ARG1, AGTR2, and GAP43. The reverse-transcribed cDNA was synthesized with the Prime-Script reagent Kit (TaKaRa, Dalian, China). PCR was performed with SYBR Green Premix Ex Taq (TaKaRa) on an Applied Biosystems Stepone real-time PCR System in triplicate for each sample. The relative mRNA level was calculated using the comparative 2^−∆∆Ct^ method and normalized against GAPDH level. All data were expressed as mean ± SD. The sequence of all primers and amplification efficiency are provided in Table S11. In addition, Pearson’s correlation analysis was used to correlate both RT-qPCR and microarray data by the aid of Commonly Used Excel Functions called CORREL. The correlation coefficients (Rs) were calculated.

## Additional Information

**How to cite this article**: Li, S. *et al.* The transcriptional landscape of dorsal root ganglia after sciatic nerve transection. *Sci. Rep.* **5**, 16888; doi: 10.1038/srep16888 (2015).

## Supplementary Material

Supplementary Table 1

Supplementary Table 2

Supplementary Table 3

Supplementary Table 4

Supplementary Table 5

Supplementary Table 6

Supplementary Table 7

Supplementary Table 8

Supplementary Table 9

Supplementary Table 10

Supplementary Table 11
